# Buffer Stock Inventory Control Mechanism: An Approach of Minimizing the Buffer Stock Level Through Segmentation at a Tertiary Care Rural Hospital

**DOI:** 10.7759/cureus.67423

**Published:** 2024-08-21

**Authors:** Rashmi Ranjan Guru, Subhodip Mitra, Sammita J Jadhav, Abdullahi K Maikano, Rahul Kumar

**Affiliations:** 1 Department of Hospital Administration, Symbiosis Institute of Health Sciences, Pune, IND; 2 Department of Hospital Administration, All India Institute of Medical Sciences, Kalyani, IND; 3 Department of Health Sciences, Symbiosis Institute of Health Sciences, Pune, IND; 4 Department of Hospital and Healthcare Management, Symbiosis Institute of Health Sciences, Pune, IND

**Keywords:** inventory control methods, buffer stock, abc-ved matrix, ved analysis, abc analysis

## Abstract

Background

Efficient management of hospital inventories, particularly pharmaceuticals, is essential for ensuring timely patient care and optimizing resource allocation. Buffer stock is the backup stock, which is kept for providing a supply of drugs when the main stock is consumed and new stock is in the process of procurement. The buffer stock is sometimes kept in excess, which causes unnecessary overutilization of the financial resources. Therefore, the author’s team aimed to optimize the inventory control of the buffer stock. This study addressed these challenges by integrating always better control (ABC) and vital, essential, and desirable (VED) analysis to categorize drugs based on consumption patterns and clinical importance to minimize buffer stock and ensure optimum resource allocation. To overcome these challenges, there is a need to integrate financial and clinical factors into inventory management decisions.

Methods

This study was done at the pharmacy of an apex hospital situated in a rural region of western India. The study aimed at the categorization of drugs that require strict inventory management control. The drugs and consumables used in the month of April 2024 in the hospital pharmacy were considered for the study & inventory control methods were applied.

Results

The study discovered that a subgroup of drugs accounted for a significant portion of the pharmacy's budget, with 12.18% categorized as high-value (Category A), 22.07% as moderate-value (Category B), and 65.74% as low-value (Category C) items. ABC analysis showed that Category A drugs consumed 70.1% of the average daily expenditure (ADE) of the pharmacy, while Category B and Category C drugs contributed 19.9% and 9.98% of the ADE, respectively. VED analysis classified drugs into vital (11.34%), essential (58.26%), and desirable (30.39%) categories. The ABC-VED matrix further categorized drugs into subgroups, with class I items constituting 20.62% of the inventory and accounting for 73.82% of the ADE. Class II items comprised 56.69% of the inventory, consuming 22.91% of the ADE, while class III items constituted 22.68% of the inventory and consumed 3.28% of the ADE.

Conclusion

ABC and VED analysis integration provides a comprehensive framework for optimizing drug inventory management in healthcare facilities. By considering both financial and clinical factors, this approach enables tailored management strategies, minimizing buffer stock, eliminating stockouts, and enhancing patient care. The rural hospital's location is responsible for maintaining the buffer stock level and the reorder level quantity. This study highlighted the role of inventory control techniques in healthcare facilities, particularly in pharmacies, to ensure the availability of essential medications while optimizing resource utilization. By integrating segmentation techniques such as ABC and VED analysis, this study provided valuable insights into categorizing drugs based on their consumption patterns, criticality, and importance in the clinical area.

## Introduction

A hospital's inventory consists of many items essential for patient treatment and care, including pharmaceuticals, medical equipment, surgical supplies, and more. Healthcare research has the goal of improving patient service levels. Healthcare facilities must always be equipped with the necessary resources to meet the ongoing demands of patients, ensuring no delays occur in treating individuals with critical health conditions. Effective management of hospital pharmacy inventory is important, particularly in developing countries such as India, where optimizing supply chains can significantly enhance healthcare delivery and patient outcomes [1]. In addition, to fulfill the regular demands, healthcare units must possess efficient resources to handle emergencies, providing optimal care to patients arriving unexpectedly in highly critical states. Maintaining adequate resources to meet regular and emergency patient demands necessitates the availability of treatment-related inventory items at the right time and place [2]. Drug shortages stem from multiple factors, compelling healthcare organizations to resort to costlier alternatives to sustain their operations effectively. Hospitals frequently use an over-provisioning mechanism known as safety or buffer stock to address these challenges [3]. Stock levels are determined based on the criticality of items, those that are highly critical are made available always. In contrast, those that are moderately critical are stocked based on cost-effectiveness, and low-critical ones are destocked. A detailed examination of pharmaceutical item data uncovered significant variations in criticality, demand, and price. Consequently, the goal is to categorize them into distinct classes to determine if different inventory control methods are necessary for each class [4]. Establishing optimal drug inventory levels is a critical step. Well-managed inventory settings are essential for maintaining stable operations and ensuring continuous drug supply [5].

There is a need to identify and prioritize drugs based on their consumption value and criticality to ensure efficient procurement practices. Despite research indicating the potential for inventory optimization models to reduce costs and maintain service levels, their adoption in healthcare settings remains limited [6].

There is a significant amount of non-critical inventory occupying space in the pharmacy. As a result, the hospital needs to allocate a substantial floor area for inventory storage and management, which can pose significant challenges. This inefficiency can lead to difficulties in inventory management, including excessive buffer stock and expired drugs. Traditionally, this emphasis has been on assembling a team of highly skilled physicians, surgeons, and proficient nurses, supported by a quality infrastructure, equipped with advanced medical technology and quality pharmaceuticals and supplies [7]. However, recent trends have shifted toward prioritizing the management aspect of healthcare systems. Various operational functions such as inventory control mechanisms, calculating reorder level, lead time, etc aimed at maximizing efficiency and effectiveness [8]. One very important inventory management issue is too much buffer stock and expiries of disaster medicines.

Drugs and consumables constitute the second highest expenditure after the wages of manpower [9]. Always better control (ABC) analysis is a widely recognized tool globally, highlighting specific items that require heightened control and management [10]. By implementing ABC and vital, essential, and desirable (VED) techniques, hospitals can reduce expenses, minimize stock shortages, and optimize inventory levels, resulting in cost savings and improved financial management. Prioritizing vital drugs through these analyses is crucial for maintaining quality healthcare services and ensuring timely and appropriate treatment for patients. Evidence-based decision-making supported by ABC and VED analysis guides procurement, distribution, and stock control strategies, enabling hospitals to make informed decisions and streamline inventory management practices [11].

Efficiently managing pharmaceutical inventory involves categorizing items and establishing specific inventory strategies for each category. It is essential to optimize and integrate supply chain management of inventory to improve the procurement management and utilization of drugs. Understanding the root causes of drug shortages is paramount for hospitals and health systems to mitigate these issues effectively [12].

This ensures a consistent approach to manage items within the same group, including service levels and replenishment policies. Traditional methods like ABC analysis, which assesses items in accordance with the yearly consumption rate, and VED classification, which evaluates how criticality of drugs, are commonly used. A study further considers factors such as treatment importance, supply characteristics, inventory challenges, and rate of consumption to determine where buffer stock should be stored in a hospital [13]. The ABC analysis categorizes drugs into Category A drugs with high value, Category B with moderate value, and Category C with low value. Some researchers contend that inventory control and planning should prioritize items based on their relative importance, through employing techniques like ABC analysis. This method categorizes items into A (high priority/impact), B (medium priority/impact), and C (low priority/impact) based on their consumption value. Other authors categorized the drugs and consumables based on their demand in the hospital i.e. high priority, mid priority and low-priority drugs [14].

Depending on only ABC classification may not adequately benefit inventory management, as it could classify an item as low priority based on low consumption values, even if it is essential for life-saving purposes. To tackle this issue, some researchers propose the use of multiple criteria for inventory classification, integrating the clinical importance of drugs by employing vital, essential, and desirable classification [15]. Both ABC and VED methods categorize drugs based on their importance, enabling the application of distinct management policies for effective drug inventory control [16].

Buffer stock is the backup stock, which is kept for providing a supply of drugs when the main stock is consumed, and new stock is in the process of procurement. The buffer stock is sometimes kept in excess which causes unnecessary overutilization of the financial resources. Therefore, the author’s team tried to optimize the inventory control of the buffer stock.

Rationale

Ensuring the availability of vital and essential drugs along with the maintenance of the buffer stock through effective inventory control is crucial for maintaining high standards of patient care and treatment outcomes. Due to the limitations of relying solely on the ABC analysis model for inventory classification, which categorizes items based on their consumption values, it may not effectively prioritize critical medications. This recognition underscores the need for additional criteria, such as the Vital, Essential, and Desirable items classification, to integrate clinical importance into inventory management decisions. Segmentation strategies are common in supply chain management, where categorizing items based on their characteristics enables tailored management strategies.

Problem statement

The persistent challenge of drug shortages poses a critical concern for healthcare systems, triggering various adverse effects. To prevent shortages in a hospital, efficient inventory management is essential for sustainable system operation. Limited resources necessitate appropriate utilization to serve more patients within budget constraints, highlighting the importance of rational drug use and improved management practices.

The authors of the study aimed to identify the medications that need stringent control for optimal fund utilization and to prevent stockouts in the pharmacy by reducing the buffer stock level for non-critical and low-consumption medications.

## Materials and methods

This study was conducted at a tertiary care hospital (Symbiosis Medical College for Women and Symbiosis University Hospital & Research Centre) based in Pune, western India, focusing on inventory management practices within its pharmacy. A purposive sampling method was utilized here, focusing on the central pharmacy of the institution in the study, and the authors tried to exclude the bias from the study. As the study place is a medical college hospital, there is one central pharmacy and four other pharmacies in different locations, like OPD, IPD, OT, and ICU. All pharmacy items are intended for the central pharmacy, received in the central pharmacy, and issued to four sub-pharmacies. That is why the central pharmacy data was considered for proper and easy calculation. The study examined data segmentation techniques such as ABC and VED analysis. For ABC analysis, the data of monthly consumption and sales costs incurred on each pharmacy item for April 2024 were included in the study. The data and the budget of the hospital are very large. The hospital is situated at a hill station in western India. The seasonal variations are not very distinguishable. In the past four years, it has been shown that the month of April has the highest number of admissions to the hospital. That is why the authors selected the month of April for easy calculation. Fast-moving consumer goods (FMCG) were excluded from the study. The data was then transcribed into an MS Excel spreadsheet. The statistical analysis was carried out using statistical functions. Drugs were arranged based on annual expenditure in descending order. The cumulative cost and percentage of expenditure for each item were calculated. Drugs were classified into A, B, and C categories based on cumulative cost percentages of 70%, 20%, and 10%, respectively. Drugs were also Classified into vital (V), essential (E), and desirable (D) categories based on criticality to care. The ABC-VED matrix was created to identify items needing stringent management control. The authors further segmented the drugs into three classes, namely Class I, Class II, and Class III. AV, AE, AD, BV, and CV were included in Class I. BE, BD, and CE were included in Class II, and CD items were included in Class III. The focus was on Category I items (vital and expensive) for strict managerial control. The data analysis plan used descriptive statistics to analyze the collected data.

Interventions

Segmentation techniques such as ABC and VED analysis were used to categorize drugs based on their consumption patterns, value, and criticality and to generate a list of items for each category.

Data analysis

The data was collected from the Hospital Information Management System (HIMS) and analyzed by using IBM SPSS Statistics for Windows, Version 21 (Released 2012; IBM Corp., Armonk, New York, United States) developed by International Business Machines Corporation (IBM). Descriptive statistics were used to summarize and describe the characteristics of the data collected from the study. Average daily consumption, cumulative frequencies, and percentages were calculated.

Ethical considerations

As this was a quality improvement process of the hospital pharmacy, no informed patient consent was required for the study.

## Results

The hospital formulary consisted of 1220 items, out of which the most used drugs and consumable items were 829, with a monetary value of ₹ 39,82,884.90. In this study, the authors found that 12% of the drugs used about 70% of the budget of the average daily expenditure of the pharmacy.

In April 2024, out of the 829 drugs, 101 (12.2%) were considered in Category A, 183 (22.07%) in Category B, and 545 (65.74%) in Category C. Category A with a monetary value of ₹ 93,074(70.1%), Category B with a value of ₹ 26,441.361(19.9%), and Category C with ₹ 13247.26(9.98%), as shown in Table 1. In April 2024, 94 (11.34%) items, 483 (58.26%) items, and 252 (30.39%) were grouped as vital, essential, and desirable categories of drugs, respectively.

**Table 1:** ABC, VED categorization, and ABC-VED Matrix Analysis of Central Pharmacy of Symbiosis Medical College for Women and Symbiosis University Hospital & Research Centre in Pune, India ABC: Always better control; VED: vital, essential, and desirable; ADE: average daily expenditure

Category	No of Items	% of Items	ADE (Rs)	% of ADE
A	101	12.18	93074.21	70.10
B	183	22.07	26441.36	19.92
C	545	65.74	13247.26	9.98
V	94	11.34	22087.49	16.64
E	483	58.26	88022.05	66.30
D	252	30.39	22653.28	17.06
I	171	20.63	98000.54	73.82
II	470	56.69	30415.37	22.91
III	188	22.68	4346.92	3.27

The authors further segmented the drugs into three classes namely Class I, Class II, and Class III. AV, AE, AD, BV, and CV were included in Class I. BE, BD, and CE were included in Class II and CD items included in Class III. Class I items made up 171 (20.62%), class II had 470 (56.69%), and Class III had 188 (22.68%), as shown in Table 1.

## Discussion

ABC segmentation techniques

**Figure 1:**
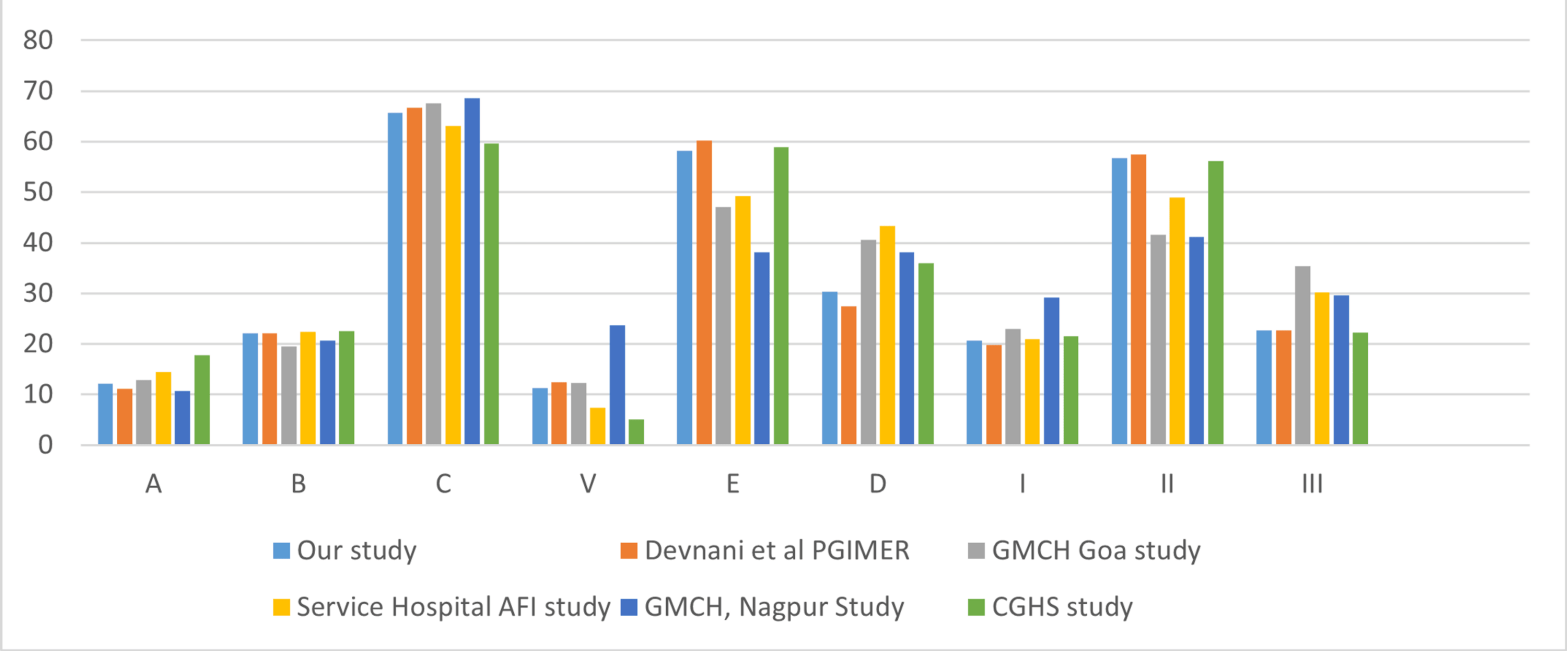
Comparison of ABC, VED segmentation and ABC-VED Matrix Analysis of the Central Pharmacy of Symbiosis Medical College for Women and Symbiosis University Hospital & Research Centre with Other Studies in India X axis: categorization and segmentation of pharmacy items; Y axis: percentage of items Source: Devnani et al. PGIMER study [11]; GMCH Goa study [15]; GMCH; CGHS study [17]; Nagpur study [19]; Service Hospital AFI study [20]

In April 2024, out of the 829 drugs from the hospital formulary, 101 (12.2%), 183 (22.07%), and 545 (65.74%) drugs were categorized into Category A, Category B, and Category C respectively having a monetary value of Rs 93,074 (70.1%), Rs 26,441.361 (19.9%), and 13247.26 (9.98%) respectively as shown in Table 1. ABC analysis done in the Central Government Health Scheme (CGHS) Pharmacy Stores revealed that 52 types (17.8%) of items used 70% of the finance as Category A, 66 (22.6%) items used 20% of the budget categorized as Category B and 174 (59.6%) items used 10% of the budget categorized as Category C [17]. The drawback of ABC analysis is that it may lead to a lack of availability of vital items categorized as B and C. The findings of our study align with those of similar research conducted in India, as shown in Figure 1. When the author's team implemented ABC analysis only, they effectively controlled drugs from Category A but compromised the availability of vital drugs from Categories B and C. The proposed approach of the ABC-VED matrix significantly reduced the effort and complexity by focusing optimization efforts on only 17.2% of the sample data and utilizing material replenishment policy strategies, lot-sizing methods, and data from the hospital's enterprise resource planning system, including economic order quantities and reorder point planning [18].

VED segmentation techniques

In April 2024, 94 (11.34%) items, 483 (58.26%) items, and 252 (30.39%) were grouped as vital, essential, and desired, respectively, as shown in Table 1. A study at a teaching hospital found that approximately 23.8 percent of drugs (53 items) were classified as vital, while another 38.1 percent (85 items) were deemed essential. The remaining 38.1 percent (85 items) were categorized as desirable [19]. If we consider VED alone, ideal control of vital and essential groups will be done, leaving some group A drugs that are in the desired category.

ABC-VED matrix

We divided the groups into nine subgroups: AV (high value, vital), AE (high value, essential), AD (high value, desired), BV (moderate value, vital), CV (low value, vital), BE (moderate value, essential), BD (moderate value, desired), CE (low value, essential), and CD. The authors further segmented the drugs into three classes namely Class I, Class II, Class III. AV, AE, AD, BV, and CV were included in Class I. BE, BD, and CE were included in Class II and CD items were included in Class III. Class I goods made up 171 (20.62%), Class II had 470 (56.69%), and Class III had 188 (22.68%) (Table 1). A study conducted in Chandigarh discovered that there were 93 (22.09%) products classified in Category I, 230 (54.63%) items in Category II, and 98 (23.28%) items in Category III. These categories accounted for 74.21% (Rs. 29,691,956), 22.23% (Rs. 8,895,160), and 3.56% (Rs. 1,425,496) of ADE in the pharmacy, respectively [11,20]. In comparison to other research in India, vital, essential, and desirable products had similar percentages (Figure 1).

Recommendations 

Category I drugs need strict managerial control due to their high cost and critical importance. These items represent an average daily expenditure of 98000.54 (73.82%) of the pharmacy’s ADE. AV, AE, and BV subgroups comprise expensive items critical to patient care, making stockouts unacceptable. Low buffer stock needs to be maintained by closely monitoring consumption levels and current stock to prevent the locking up of capital due to these items.

CV category drugs are low-cost but vital drugs, accounting for a negligible portion of the ADE; hence, these items can be procured annually due to their low carrying cost. AD items account for 2.77% of the total drug items. They are costly and desirable, requiring careful economic order quantity management. Buffer stock for these drugs is not required as they have a high carrying cost. Items from this category if removed from the hospital formulary, there will be substantial monetary savings. The drugs are low-cost but critical, so their procurement and stocking are done annually to reduce the ordering cost, as the hospital is a rural hospital situated far away from the town where the drug vendors or suppliers are located. High-cost and critical drugs are being procured from local vendors with rate contracts. For the overall saving of procurement costs and carrying costs, a calculated minimum suitable buffer stock for the short repetitive period is maintained.

Category II items (BE, BD, and CE) consume 22.91% of ADE (30415.37). The category of drugs is essential, and their need is always in the hospital. but it constitutes only 22% of the budget. As the hospital's location is a matter of concern, and to reduce the ordering or transportation cost, it is advised to reduce their orders and make the drugs always available for the patient. Ordering these items once or twice annually can save costs and reduce management complexity, despite their moderate carrying costs. Therefore, the buffer stock of this category is recommended to be kept at a minimum level. 

Category III (CV) items consume 3.28% of ADE (4346.92). These items are recommended to have a re-order level in once or twice a year thus saving the ordering cost. This helps in saving management efforts, ensuring year-round availability, without locking substantial capital in the stock-carrying cost, which resulted in less management strategy to optimize resources and continuous availability in the hospital pharmacy. This group of drugs is also recommended to be kept at a low level of buffer stock.

Limitations

This study was conducted at a single healthcare facility, limiting the findings to different levels of healthcare setups, availability of resources, and patient demographics. The situatedness of the hospital is the main contributory factor for the maintenance of the buffer stock. The data and the budget of the hospital are very large. The hospital is situated in a rural hill station. The seasonal variations are not very distinguishable. One-month data considered for the study seem to be a limitation of the study.

## Conclusions

The rural hospital's location is responsible for maintaining the buffer stock level and the reorder level quantity. This study highlighted the role of inventory control techniques for the maintenance of buffer stock in healthcare facilities, particularly pharmacies, to ensure the availability of essential medications while optimizing resource utilization. By integrating segmentation techniques such as ABC and VED analysis, this study provided valuable insights into categorizing drugs based on their consumption patterns, criticality, and importance in the clinical area, thereby setting the buffer stock level. The findings highlighted the significance of employing a multi-dimensional approach, as relying solely on ABC analysis might overlook vital medications categorized as moderate or low value. Conversely, VED analysis alone may prioritize clinical importance but overlook financial considerations, leading to inefficient allocation of resources. This approach enabled more effective resource allocation and inventory management prioritization by integrating financial considerations (ABC analysis) and clinical criteria (VED analysis). Through the ABC-VED matrix, this study proposes a comprehensive framework that accounts for both financial and patient care factors for maintaining the buffer stock level at an optimum level and making standard operative procedures for the just-in-time techniques.
